# Supplementary material for: A dual role for the RNA helicase DHX34 in NMD and pre-mRNA splicing and its function in hematopoietic differentiation
Source: RNA. 2022 Sep;28(9):1224–38. doi: 10.1261/rna.079277.122 (PMC9380745; doi:10.1261/rna.079277.122)
Supplement: Supplemental Material [file supp_28_9_1224__DC1.html]

A dual role for the RNA helicase DHX34 in NMD and pre-mRNA splicing and its function in hematopoietic differentiation — Supplemental Material 

# A dual role for the RNA helicase DHX34 in NMD and pre-mRNA splicing and its function in hematopoietic differentiation

## Supplemental Material

- Supplemental\_Figures.docx
- Supplemental\_Table\_1.xlsx
- Supplemental\_Table\_2.xlsx
- Supplemental\_Table\_3.xlsx
- Supplemental\_Table\_4.docx
